# Hepatoprotective Mechanism of Ginsenoside Rg1 against Alcoholic Liver Damage Based on Gut Microbiota and Network Pharmacology

**DOI:** 10.1155/2022/5025237

**Published:** 2022-08-23

**Authors:** Ting Xia, Bin Fang, Chaoyan Kang, Yuxuan Zhao, Xiao Qiang, Xiaodong Zhang, Yiming Wang, Tian Zhong, Jianbo Xiao, Min Wang

**Affiliations:** ^1^State Key Laboratory of Food Nutrition and Safety, Key Laboratory of Industrial Fermentation Microbiology, College of Biotechnology, Tianjin University of Science and Technology, Tianjin 300457, China; ^2^Faculty of Medicine, Macau University of Science and Technology, Taipa, Macau 999078, China; ^3^Universidade de Vigo, Department of Analytical and Food Chemistry, Faculty of Sciences, 32004 Ourense, Spain; ^4^International Research Center for Food Nutrition and Safety, Jiangsu University, Zhenjiang 212013, China

## Abstract

Alcoholic liver disease (ALD) is a major public health problem worldwide, which needs to be effective prevention. Ginsenoside Rg1 (GRg1), a bioactive ingredient extracted from ginseng, has benefit effects on health. In this study, 11 potential targets of GRg1 against ALD were firstly obtained by network pharmacology. KEGG pathway enrichment showed that GRg1-target-ALD was closely related to Toll-like receptor (TLR) and nuclear factor-kappa B (NF-*κ*B) signaling pathways. In addition, GRg1 decreased antioxidant levels and increased oxidative levels in alcohol-treated mice, which alleviated oxidative stress-induced hepatic damage. GRg1 enhanced intestinal barrier function *via* upregulating the levels of tight junction protein and immunoglobulin A. GRg1 also reduced alcohol-induced inflammation by suppressing TLR4/NF-*κ*B pathway, which was consistent with the prediction of network targets. Moreover, GRg1 altered GM population, and Verrucomicrobia, Bacteroidetes, *Akkermansia*, *Bacteroides*, *Lachnospiraceae_NK4A136_group*, and *Alloprevotella* played positive association with intestinal barrier indicators and negative correlation with hepatic inflammation biomarkers. The results suggest that GRg1 administration might be a promising strategy for protection of alcohol-induced liver damage.

## 1. Introduction

Alcoholic liver disease (ALD) is an ubiquitous health burden around the world, which causes progression of liver damage [1, 2]. It has been demonstrated that oxidative stress is a major pathogenesis of alcohol-induced liver damage, which is closely related to the development of ALD [3]. Alcohol administration induces reactive oxygen species (ROS) generation and oxidative products, which leads to the destruction of antioxidative system [4]. Previous study has reported that excess ROS induced by alcohol can activate Kupffer cells and produce proinflammatory factors [5].

Emerging studies have proved that the alteration of gut microbiota (GM) is a causative factor in ALD [6, 7]. Meanwhile, excessive alcohol intake destroys the integrity of intestinal barrier and promotes lipopolysaccharides (LPS) releasing from intestine to liver through blood circulation [8]. Toll-like receptor 4 (TLR4) is activated by LPS in Kupffer cells and induces the expression of p-nuclear factor-kappa B (p-NF-*κ*B). TLR4/nuclear factor-kappa B (NF-*κ*B) pathway results in the release of inflammatory factors, which subsequently contributes to liver damage [9]. Therefore, alteration of GM and inhibition of inflammatory could be an effective strategy to prevent alcohol-induced liver injury.

Ginsenosides are bioactive compounds extracted from *Panax ginseng* C.A. Meyer, which have many pharmacological properties including anti-inflammation, antioxidant activity, and anticancer [10, 11]. Among these ginsenosides, ginsenoside Rg1 (GRg1) accounts for about 0.22% in sun-cured ginseng and Radix ginseng rubra, which is a major bioactive component [12]. Recent studies reported that GRg1 displayed remarkable antioxidant activity and exhibited protection of liver injury [13, 14]. However, the effect and mechanism of GRg1 on GM in mice with alcoholic liver damage remain unclear.

Recently, network pharmacology is used to estimate the molecular mechanism of drugs from multiple dimensions. It reveals the potentially complex relationship between drugs and their targets according to “disease-target-ingredient-drug” network model [15]. This method has been successfully used in the research of Chinese herbal medicine, especially in seeking of bioactive ingredients and their therapeutic targets [16]. Therefore, network pharmacology can provide a valid strategy to further explore the potential targets of GRg1 to prevent ALD.

In this study, the potential ALD targets of GRg1 were predicted through network pharmacology firstly. The effects of GRg1 on ethanol-induced liver injury were investigated *in vivo*. The potential targeted pathway was explored to verify the analysis of network pharmacology. Moreover, GM composition and gut barrier were explored in the intestine. GM interplayed with host indexes was estimated in ethanol-treated mice. The findings would provide an alternative agent from ginsen*g* for ALD prevention.

## 2. Materials and Methods

### 2.1. Materials

**Figure 1:**
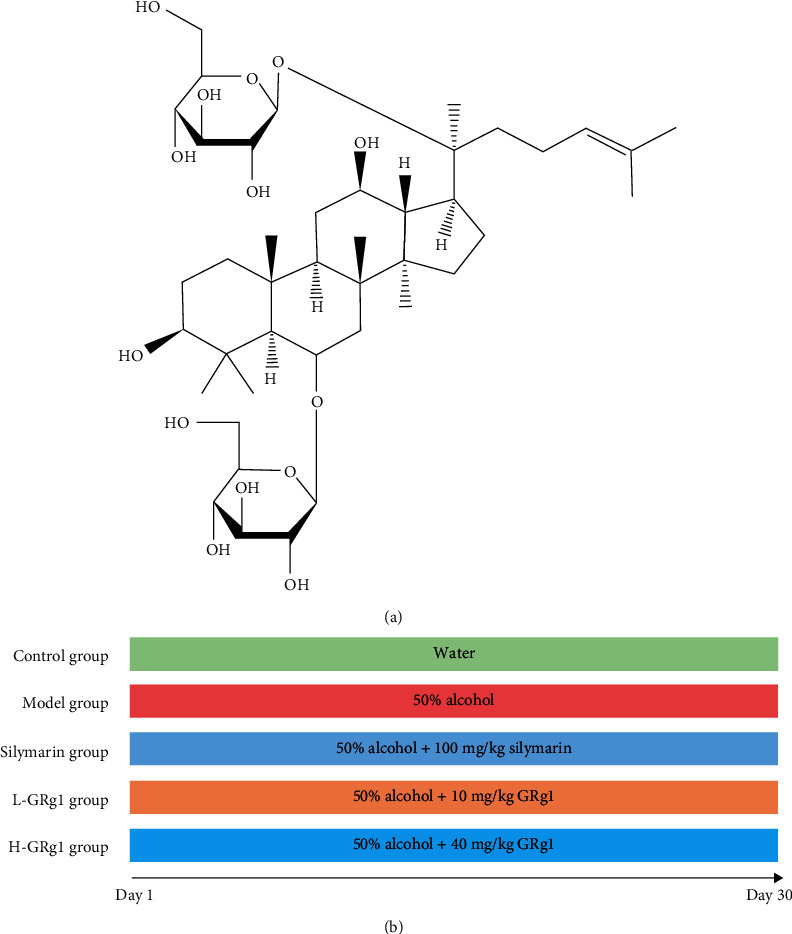
Chemical structure of GRg1.

GRg1 (purity ≥ 98%) was obtained from Beijing Beina Chuanglian Biotechnology Technology Research Institute (Beijing, China), which was dissolved with distilled water. The structure of GRg1 is shown in Figure 1. Silymarin was purchased from Madaus AG. (Cologne, Germany), which was dissolved with olive oil. The primary antibodies against TLR4 (Santa Cruz Biotechnology, Santa Cruz, CA, USA) and p-NF-*κ*B p65 (Abcam, Cambridge, UK), and the second antibody (Cell Signaling Technology, Danvers, MA, USA) was purchased.

### 2.2. Network Pharmacology Analysis

The information of bioactive ingredients of ginseng was obtained from Traditional Chinese Medicine Systems Pharmacology Database (TCMSP). The corresponding targets of these ingredients were screened through HERB (http://herb.ac.cn/), TCMSP, and literature retrieval. In addition, ALD as the key word was screeched in the GeneCards databases (https://www.genecards.org/). The targets related to ALD were analyzed according to relevance score ≥ 40. All targets were converted into their gene names by Uniport database.

The targets of bioactive components in ginseng associated with ALD were determined by R x64 3.6.3 and represented in Venn diagrams. The network analysis of herb-ingredient-target-disease was visualized by Cytoscape 3.6.1. The shared targets were upload to STRING for analysis of protein-protein interaction (PPI), and then, protein type was set as Homo Sapiens. The data were saved and enriched by R x64 3.6.3. Meanwhile, the clusterProfiler package in R x64 3.6.3 was used to label and visualize KEGG pathway, to predict the pathway expression of these targets.

### 2.3. Design of Animal Experiments

Forty male ICR mice (6 weeks old, 18-22 g) were provided by Beijing Vital River Laboratory Animal Technology Co., Ltd. (Beijing, China). The pathogen-free environment for raising experimental mice was provided by the Animal Committee of Nankai University (SYXK2019-0001, permission date: Jan 11, 2019). We conducted animal experiments following animal ethics. The schematic diagram of animal experimental protocol was shown in Figure S1. The dose of GRg1 administered by gavage was 10 mg/kg body weight (b.w.) and 40 mg/kg b.w. [17, 18]. ICR mice were randomized into the groups of control, alcohol, alcohol + silymarin (100 mg/kg b.w.), alcohol + low − dose GRg1 (10 mg/kg b.w.), and alcohol + high − dose GRg1 (40 mg/kg b.w.). The control group was orally administrated with distilled water, and the alcohol group was given by gavage with alcohol (Sigma-Aldrich, St. Louis, MO, USA) for 30 days with an increased dose (2-6 g/kg b.w.). Silymarin (100 mg/kg b.w.) and GRg1 (10 and 40 mg/kg b.w., respectively) were given to alcohol + silymarin and alcohol + GRg1 groups and then to alcohol by daily gavage 2 h later. After 12 h, the mice were anesthetized with sodium pentobarbital (50 mg/kg, i.p.) and euthanized by cervical dislocation. The serum in each mouse was obtained by blood centrifugation, and then, the liver and intestinal tissues of the mice were immediately placed in refrigerator (-80°C).

### 2.4. Histopathological Observation

The liver and colon tissues were removed by laparotomy and immersed in formaldehyde solution. Tissues were embedded in paraffin wax. Then, the tissue samples were cut into 5-8 *μ*m thickness and stained by hematoxylin-eosin (H&E) and Alcian blue-periodic acid Schiff (AB-PAS), respectively. The degree of inflammation and lipid accumulation of in these tissues was observed.

### 2.5. Analysis of Biochemical Indexes

The circulating levels of alanine aminotransferase (ALT), aspartate aminotransferase (AST), lactate dehydrogenase (LDH), and alkaline phosphatase (AKP) were measured by detection kits (Nanjing Jiancheng Bioengineering Institute, Nanjing, China). Hepatic levels of malondialdehyde (MDA), glutathione peroxidase (GSH-Px), glutathione (GSH), catalase (CAT), and superoxide dismutase (SOD) were detected by detection kits of Nanjing Jiancheng Bioengineering Institute.

### 2.6. Enzyme-Linked Immunosorbent Assay (ELISA)

The liver and colon tissue samples were grinded into homogenate. The sample supernatant of serum, liver, and colon was obtained after centrifugation. The total protein contents were measured by a BCA protein kit. The levels of LPS, tumor necrosis factor *α* (TNF-*α*), interleukin-1 beta (IL-1*β*), interleukin-6 (IL-6), transforming growth factor-*β*1 (TGF-*β*1), ROS, 8-hydroxy-2′-deoxyguanosine (8-OHdG), 4-hydroxynonenal (4-HNE), and immunoglobulin A (IgA) in each sample were measured by ELISA reader (Tecan, Salzburg, Austria).

### 2.7. Analysis of Reverse Transcription-Quantitative Polymerase Chain Reaction (RT-qPCR)

The extraction kit (Promega Corporation, Madison, WI, USA) was used to extract the total RNA of colonic tissue, which were transcribed into cDNA. The concentrations of RNA were measured by NanoPhotometer™ P300 spectrophotometer (Implen, Munchen, Germany). GAPDH gene primers were selected as internal reference genes to determine the mRNA levels of zonula occludens-1 (ZO-1), occludin, and claudin. Sequences of primers were placed in Table 1. SYBR Green PCR Master Mix kit (Vazyme Biotech Co., Ltd Nanjing, China) was applied to carry out PCR reaction. The conditions of reaction were 5 min 95°C, 10 s 95°C, 30 s 60°C, and 40 cycles. 2-*ΔΔ*Ct method was used to measure the relative mRNA expression.

### 2.8. Western Blotting Analysis

The total protein content in each sample of liver and colon was determined. Separation of proteins from different samples was separated by sodium dodecyl sulfate-polyacrylamide gel electrophoresis (10-15%) and then transferred to polyvinylidene fluoride (PVDF) membrane. The membranes were sealed and incubated with the primary antibodies and then washed and incubated with secondary antibodies at 25°C. Finally, protein expression of membranes was presented by an odyssey infrared imaging system.

### 2.9. DNA Sequencing of GM and Bioinformatic Analysis

The cecal contents of each sample were obtained in a super-clean worktable. The total microbial DNA of cecum contents was extracted by a DNA kit (Omega Biotek, Norcross, GA, USA), and then, DNA purity was evaluated. The hypervariable region (V3-V4) of 16S rRNA was multiplied by PCR technique. The purified amplicons were quantitatively analyzed and sequenced by Illumina MiSeq platform (Illumina, San Diego, CA, USA).

### 2.10. Statistical Analysis

All the results in the experiment were expressed as mean ± standard deviation (S.D.) and analyzed using GraphPad Prism 8.0. Pearson correlation coefficient and the heat map between gut microbiota and the related biomarkers were obtained on the platform of Genedenovo Biotechnology Co. Ltd (https://www.omicsmart.com/). The significant difference was calculated according to the student's *t*-test. Values of *P* < 0.05 represent statistically difference.

## 3. Results

### 3.1. Shared Targets between Bioactive Ingredients in Ginseng and ALD

**Figure 2:**
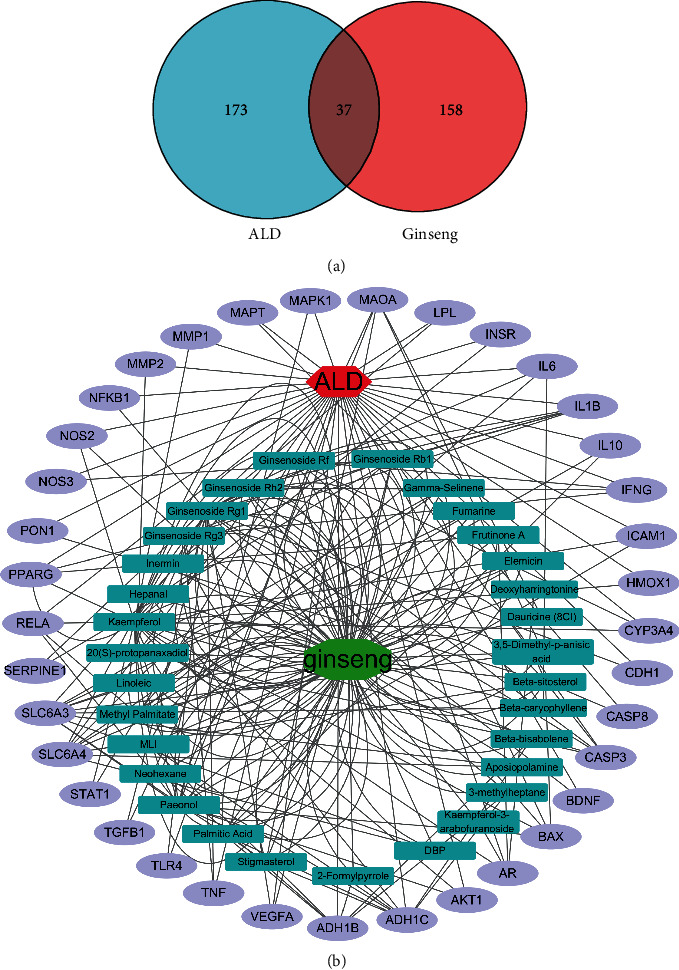
Shared targets between ginseng and ALD. (a) Venn diagram of candidate targets in ginseng and ALD. (b) Bioactive ingredients-targets-ALD network. The green octagon node represents ginseng. Cyan rectangle nodes represent the bioactive components of ginseng. The red hexagon node represents ALD. Purple ellipse nodes represent potential targets of ginseng against ALD.

The bioactive ingredients of ginseng and ALD-related targets were gathered by using the corresponding database. The results showed that 195 targets corresponding to bioactive ingredients of ginseng were obtained from TCMSP, HERB, and literatures. Meanwhile, the ALD-related genes were gathered, and 210 ALD-related targets were confirmed (relevance score ≥ 40). 37 shared targets between active ingredients and ALD-related targets were identified in generating Venn diagram (Figure 2(a)). Cytoscape software was employed to construct the network of “bioactive ingredients-targets-disease” network about common targets in schematic diagram. 31 bioactive components in Panax ginseng were associated with 37 shared targets, which have potential preventive effects on ALD (Figure 2(b)).

### 3.2. Potential Target Analysis of GRg1 against ALD

**Figure 3:**
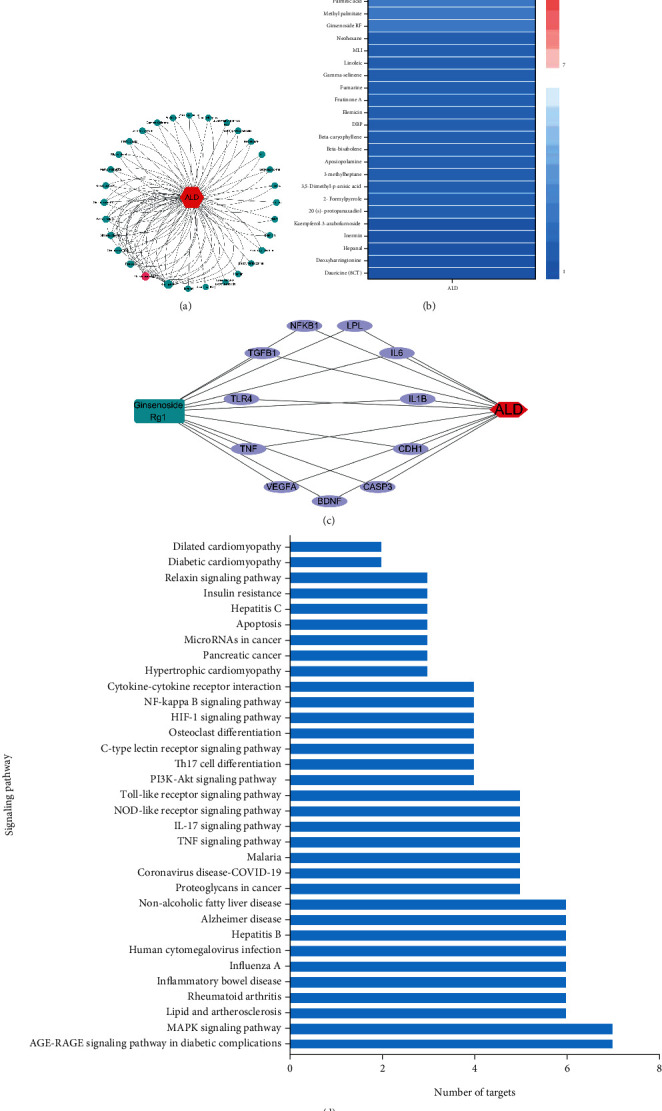
Potential targets analysis of bioactive components in ginseng against ALD. (a) Rank diagram of the degree between bioactive ingredients and ALD. The size of circle reflects the degree. (b) Heat map of the degree between bioactive ingredients and ALD. Blue and red colors indicate low and high degree value, respectively. (c) GRg1 targets ALD network. Purple ellipse nodes represent potential targets of GRg1 against ALD. (d) Enrichment diagram of KEGG pathway.

To further investigate the potential anti-ALD property of 31 bioactive ingredients, the network of bioactive components in Panax ginseng targeted to ALD was analyzed by Cytoscape software (Figure 3(a)). The degree between bioactive ingredients and ALD was visualized in heat map (Figure 3(b)). The results showed that the degree between GRg1 and ALD was ranked the second, which showed powerful ability in preventing alcoholic liver injury (Figures 3(a) and 3(b)). Recent studies have reported that GRg1 is a major ingredient originated from Panax ginseng, which has the protective effect on liver disease [16, 19]. Then, GRg1-target-ALD network was established to explore the potential targets in preventing ALD. We found that 11 genes such as IL-6, TLR4, TNF, and CASP3 were interactive targets between GRg1 and ALD, suggesting that GRg1 protected alcoholic liver damage through these targets (Figure 3(c) and Table 2). Furthermore, KEGG pathway analysis was performed on these interactive targets in Figure 3(d). The results showed that GRg1 represents its protective effects against ALD were closely related to these signaling pathways including TLR signaling pathway and NF-*κ*B signaling pathway.

### 3.3. Effect of GRg1 on Alcoholic Liver Damage in Mice

**Figure 4:**
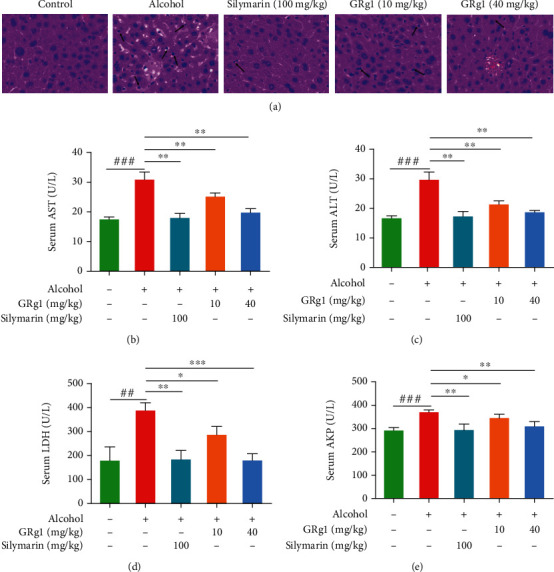
Effect of GRg1 on alcoholic liver damage *in vivo*. (a) The liver tissues were stained by hematoxylin-eosin (200×). The arrows indicate inflammatory cells. (b–e) The circulating levels of ALT, AST, LDH, and AKP. ^##^*P* < 0.01 and ^###^*P* < 0.001 vs. control group. ^∗^*P* < 0.05, ^∗∗^*P* < 0.01, and ^∗∗∗^*P* < 0.001 vs. alcohol group.

To explore the potential effects of GRg1 on ALD, the hepatic tissue morphology and plasma biochemical indexes were determined in alcohol-induced liver damage mice. As shown in Figure 4(a), the liver lobule structure was clear, and the hepatocytes were arranged regularly in control group, whereas cellular swelling and inflammatory infiltration were exhibited in the alcohol group. After pretreatment with different doses of GRg1, infiltrations of inflammatory cells were gradually decreased. The improvement of histopathology in high dose-GRg1 group was similar to that in silymarin group. Meanwhile, alcohol treatment obviously elevated the activities of hepatic enzymes including ALT, AST, LDH, and AKP in the serum. However, the enzymatic activities were gradually reduced by GRg1 pretreatment. These biochemical index in high dose-GRg1 group was similar to those in positive control group (Figures 4(b)–4(e)), indicating that the protective effect of high-dose GRg1 on alcoholic liver injury closely resembled that of silymarin. Our results demonstrate that GRg1 has protective effects on liver injury induced by alcohol.

### 3.4. Effect of GRg1 on Hepatic Oxidative Stress Induced Alcohol in Mice

**Figure 5:**
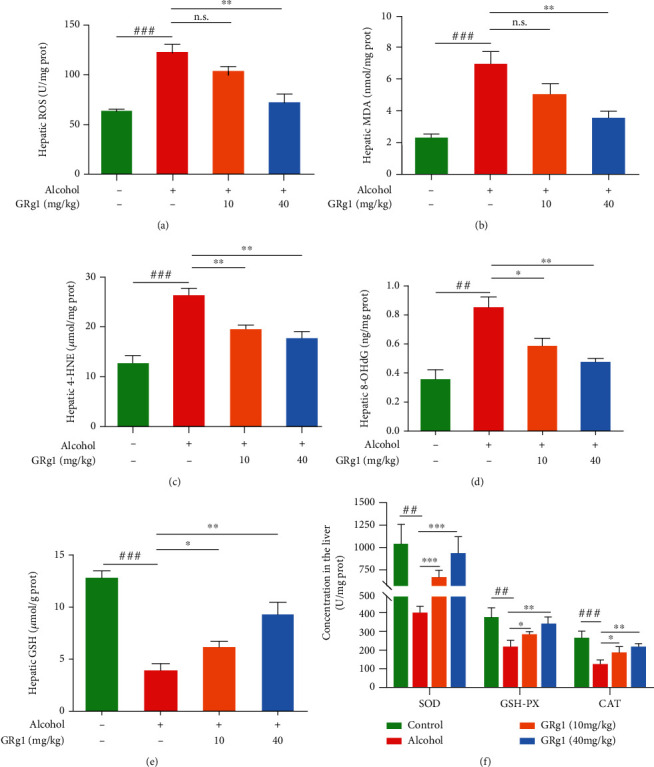
The levels of oxidative and antioxidative indexes in the liver. (a) ROS, (b) MDA, (c) 4-HNE, (d) 8-OHdG, and (e) GSH were oxidative parameters. (f) Hepatic SOD, GSH-Px, and CAT were antioxidative parameters. ^##^*P* < 0.01 and ^###^*P* < 0.001 vs. control group. ^∗^*P* < 0.05, ^∗∗^*P* < 0.01, and ^∗∗∗^*P* < 0.001 vs. alcohol group. n.s. indicates no significant difference.

Alcohol induces ROS generation and interferes with antioxidant defense system, which further results in oxidative stress in liver. Alcohol-induced oxidative stress demonstrated an essential role in promoting ALD development [20]. To investigate the effect of GRg1 on hepatic oxidative stress, the levels of oxidation and antioxidant parameters were detected. Alcohol administration significantly increased ROS level and oxidative products (MDA, 4-HNE, and 8-OHdG). However, these levels of oxidative indexes were gradually decreased in GRg1 group (Figures 5(a)–5(d)). Similarly, as shown in Figures 5(e) and 5(f), GRg1 pretreatment significantly alleviated the alcohol-induced decrease in antioxidant indices (SOD, GSH-Px, CAT, and GSH). Collectively, GRg1 prevents alcohol-induced oxidative stress by regulating the equilibrium between oxidation and antioxidation in the liver of mice.

### 3.5. GRg1 Alleviated Inflammatory Response in the Liver Treated by Alcohol

**Figure 6:**
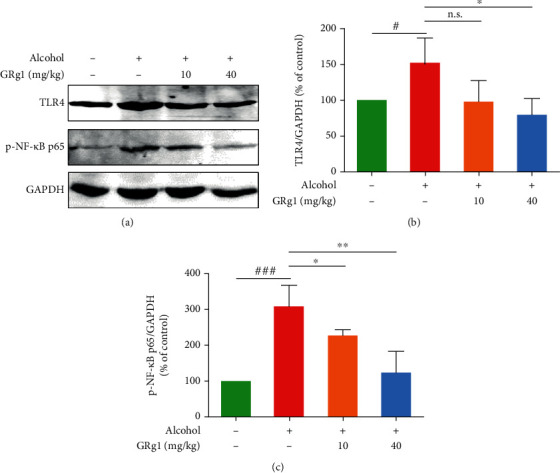
The TLR4/NF-*κ*B pathway in the liver. (a) The protein levels of TLR4 and p-NF-*κ*B p65 were detected using western blotting. Densitometric analysis of (b) TLR4 and (c) p-NF-*κ*B p65. ^#^*P* < 0.05 and ^###^*P* < 0.001 vs. control group. ^∗^*P* < 0.05 and ^∗∗^*P* < 0.01 vs. alcohol group. n.s. indicates no significant difference.

To assess the effect of GRg1 on hepatic inflammation, inflammatory indexes were measured on the basis of the network pharmacology analyses. In Table 3, LPS level was significantly elevated by alcohol. However, the elevation of LPS level in alcohol group was gradually restored by GRg1 pretreatment. Numerous studies demonstrate that low LPS concentration can activate LPS-mediated TLR4/NF-*κ*B pathway, which is essential to hepatic inflammation [21]. Then, the protein expression levels of TLR4 and p-NF-*κ*B p65 were detected through western blotting. We found that alcohol remarkably upregulated TLR4 and p-NF-*κ*B p65 expressions. However, these levels were gradually decreased after GRg1 pretreatment (Figures 6(a)–6(c)), suggesting that LPS-induced TLR4/NF-*κ*B activation is attenuated by GRg1. Furthermore, inflammatory factor levels including TNF-*α*, IL-1*β*, IL-6, and TGF-*β*1 were significantly upregulated by alcohol treatment, while GRg1 gradually decreased those levels, especially in high-dose GRg1 group (Table 3). The data imply that GRg1 inhibits LPS/TLR4/NF-*κ*B signaling pathway, which subsequently ameliorates liver inflammation induced by alcohol.

### 3.6. Effect of GRg1 on Intestinal Barrier in Alcohol-Treated Mice

**Figure 7:**
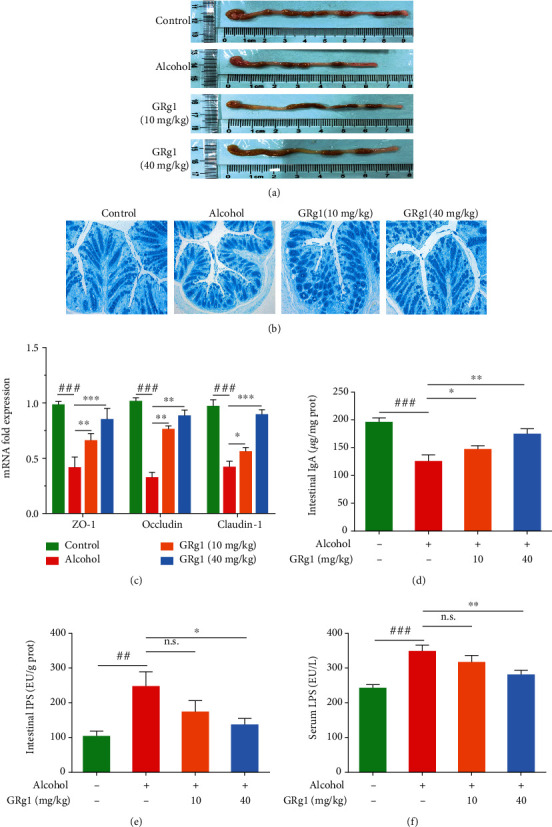
Intestinal barrier function in the colon. (a) The length of the colon. (b) The colon tissues were stained by Alcian blue (200×). (c) The mRNA expression levels of tight junction proteins (ZO-1, occludin, and claudin-1). (d) IgA levels, (e) intestinal LPS levels, and (f) circulating LPS levels. ^##^*P* < 0.01 and ^###^*P* < 0.001 vs. control group. ^∗^*P* < 0.05, ^∗∗^*P* < 0.01, and ^∗∗∗^*P* < 0.001 vs. alcohol group. n.s. indicates no significant difference.

To investigate the integrity of intestinal epithelial cells, anatomical and histopathological observations were performed in the colon. Histopathological examination showed that the shortened length of intestine was reversed by GRg1 pretreatment (Figure 7(a)). In addition, epithelial cells were destroyed and loosely lined, and the space of subepithelia was expanded in alcohol-treated mice, whereas those were obviously alleviated in GRg1 group (Figure 7(b)). Furthermore, the tight junction proteins (ZO-1, occludin, and claudin-1), as indicators to measure intestinal permeability, maintain the integrity of intestinal barrier [9]. Next, the mRNA levels of colon tight junction proteins were detected by RT-qPCR. The levels of ZO-1, occludin, and claudin-1 detected by PCR in alcohol group were gradually reduced in alcohol group, which were reversed after GRg1 pretreatment (40 mg/kg b.w.) (Figure 7(c)). Meanwhile, GRg1 pretreatment gradually restored the decrease of IgA level by alcohol (Figure 7(d)). And compared with alcohol group, the intestinal and circulating levels of LPS in the GRg1 group were reduced in a form of measurement dependence (Figures 7(e) and 7(f)). The data indicate GRg1 enhances gut barrier by promoting the expressions of tight junction protein and IgA.

### 3.7. Effect of GRg1 on the Intestinal Microbiota Composition in Alcohol-Treated Mice

**Figure 8:**
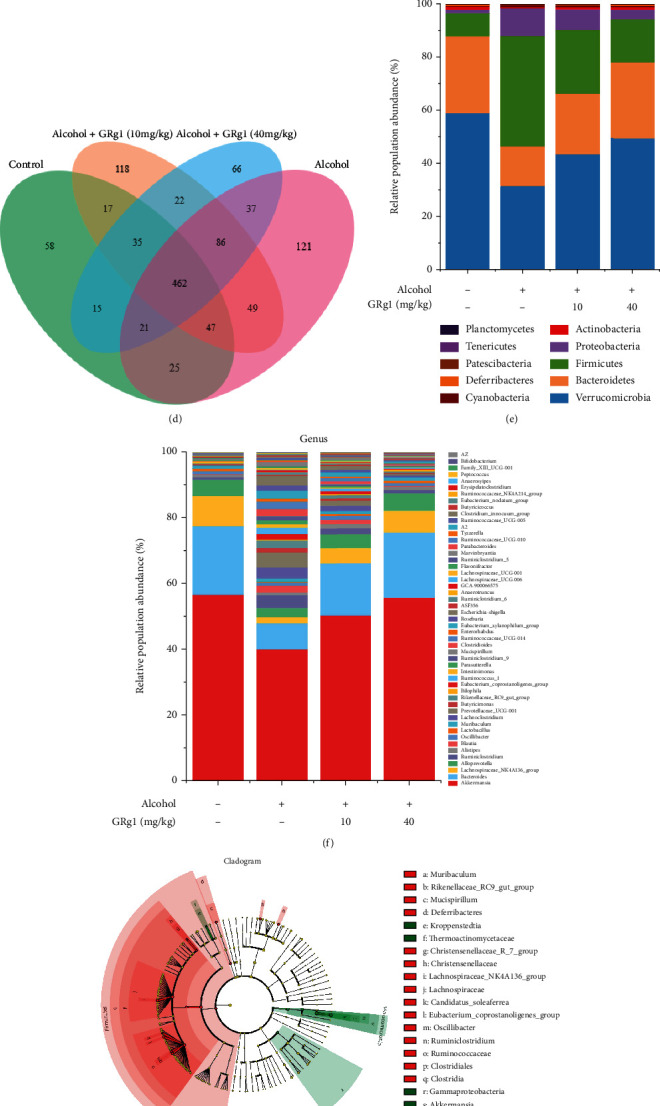
Intestinal microbiota composition in cecum. (a) Chao1 richness index. (b) Shannon diversity index. (c) PCA plot. (d) Venn diagram. (e) Relative abundances of microbial composition at phylum levels. (f) Relative abundances of microbial composition at genus levels. (g) LEfSe taxonomic cladogram between alcohol group and high-dose GRg1 group. The size of the circles is based on relative abundance. (h) LDA score diagram. ^###^*P* < 0.001 vs. control group. ^∗∗^*P* < 0.01 and ^∗∗∗^*P* < 0.001 vs. alcohol group.

To analyze the effect of GRg1 on the GM composition in ALD mice, 16S rRNA were multiplex sequenced in the intestine. In this study, after alcohol treatment, the indexes of Chao1 richness and Shannon diversity were gradually elevated, while GRg1 pretreatment obviously reduced the indexes of Chao1 richness and Shannon diversity compared with those in alcohol group (Figures 8(a) and 8(b)). As shown in Figure 8(c), the cluster of GM in alcohol group was obviously different with the clusters of normal mice, while those between GM in high-dose GRg1 group and control group were close to each other. Meanwhile, OTUs in each group were shared and different in the Venn diagram (Figure 8(d)).

Then, the different GM phylotypes among all groups were analyzed at different levels. The decrease of Verrucomicrobia and Bacteroidetes in alcohol group were reversed by GRg1 treatment (Figure 8(e)). In addition, *Akkermansia*, *Bacteroides*, *Lachnospiraceae_NK4A136_group*, and *Alloprevotella* were the most abundant genera (Figure 8(f)). To further investigate the specific differences of intestinal microbiota at various phylogenetic levels, LEfSe was used to analyze the intestinal microbiota between alcohol group and GRg1 (40 mg/kg) group. As shown in Figures 8(g) and 8(h), Firmicutes phylum, two genera including *Lachnospiraceae_NK4A136_group*, and *Ruminiclostridium* were abundant in alcohol group, while Verrucomicrobia phylum and *Akkermansiawere* genus were predominant in GRg1 (40 mg/kg) group. These results indicate that GRg1 can regulate the composition of GM in alcohol group.

### 3.8. Correlations between GM and the Indexes

**Figure 9:**
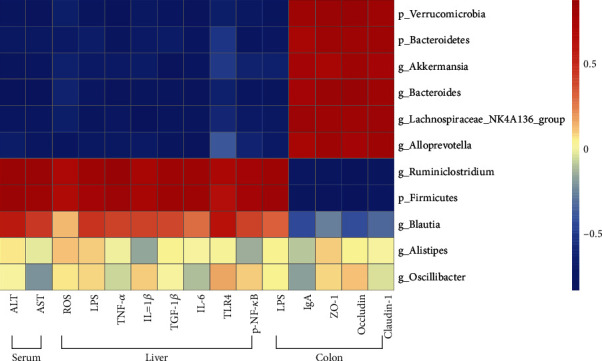
Heat map of Pearson correlation between GM and the body indexes. Blue and red colors indicate negative and positive correlation, respectively.

In order to illuminate the potential mechanisms of GRg1 on ALD, the relationship between intestinal flora and host indexes was analyzed. As shown in Figure 9, the Pearson correlation analysis showed that Verrucomicrobia, Bacteroidetes, *Akkermansia*, *Bacteroides*, *Lachnospiraceae_NK4A136_group*, and *Alloprevotella* showed obvious positive correlation with intestinal integrity indexes (tight junction proteins and sIgA), while negative correlation with hepatic biomarkers (ROS, ALT, and AST), hepatic indexes of inflammation (LPS, TNF-*α*, IL-1*β*, IL-6, and TGF-*β*1) and TLR4/NF-*κ*B expression. In contrast, Firmicutes are positively associated with these oxidative and inflammatory parameters, whereas negatively correlated with hepatic antioxidant parameters and intestinal integrity indexes. Here, the results suggest that GRg1 causes the alteration of GM population, which enhances intestinal integrity and arrests gut-derived inflammation by inhibition of LPS-mediated TLR4/NF-*κ*B pathway.

## 4. Discussion

ALD is caused by alcohol abuse, which is accompanied by the development of liver injury [22]. Oxidative stress and inflammation induced by alcohol are participated in the progression of ALD. Recent studies reported that GM participated in the evolution of alcohol-induced liver injury, which is a critical element for prevention of ALD [6, 7]. GRg1 extracted from ginseng can suppress oxidative stress and inflammatory responses, which exerts its pharmacological property to prevent and treat inflammation-related diseases [13, 19]. In this study, the potential targets of GRg1 against ALD were predicted through network pharmacology, and the underlying mechanisms of GRg1 on alcoholic liver injury were investigated in mice.

Network pharmacology is an effective approach to predict the targets and pathways of drugs, which provides a way of the precise prevention and treatment of disease [15]. In this study, 31 bioactive ingredients in ginseng were selected to have potential anti-ALD activity by ginseng-targets-ALD network (Figures 2(a) and 2(b)). Among these bioactive compounds, GRg1 showed the powerful pharmacology against ALD according to the degree values between bioactive ingredients and ALD (Figures 3(a) and 3(b)). Recent studies demonstrated that GRg1 can protect against some liver diseases in several animal models [23, 24]. Combined with the related references, GRg1 was chosen to perform the network of GRg1-tagets-ALD. We found that 11 key genes were related to GRg1, which were strongly correlated with ALD (Figure 3(c) and Table 2). In addition, KEGG pathway analysis showed the TLR and NF-*κ*B signaling pathways were interrelated with GRg1 against ALD (Figure 3(d)). It has been confirmed that the activation of LPS/TLR4/NF-*κ*B pathway induces the secretion of inflammatory factors, which contributes to the development of ALD [9, 25]. These results indicate that LPS/TLR4/NF-*κ*B pathway is the potential targets of GRg1 to protect against ALD.

Excessive alcohol consumption causes histopathological changes and disfunction in the liver [2]. The elevation of hepatic enzymes in the serum indicates the occurrence of liver injury, such as AST, ALT, and LDH [26]. It has been verified that silymarin exhibits protective effects against liver injury, which is used as a common positive control in many studies of hepatoprotective drugs [27, 28]. In the current study, it was found that GRg1 effectively alleviated histopathological changes in liver tissue and the levels of hepatic enzymes (AST, ALT, LDH, and AKP) in serum (Figure 4), indicating that GRg1 can reduce alcohol-induced liver damage. In addition, hepatoprotective effects of high dose-GRg1 were similar to silymarin, suggesting that GRg1 is a potential agent for preventing ALD. Alcohol-induced excess ROS triggers oxidative damage of lipid and DNA and destroys equilibrium of oxidation and antioxidation, which leads to hepatic inflammation and injury [4, 29]. Our results showed that GRg1 pretreatment reversed the increase of oxidative level (ROS, MDA, 4-HNE, and 8-OHdG) and decrease of antioxidative level (SOD, CAT, GSH-Px, and GSH) (Figure 5), suggesting the alleviation of alcohol-induced oxidative stress.

Several studies indicate that alcohol and its metabolites destroy the function and structure of gut epithelial cells and result in the enlargement of gut permeability [30–32]. In this study, gross and tissue staining displayed that high-dose GRg1 pretreatment alleviated the reduction of intestinal length and histopathological changes in the colon (Figures 7(a) and 7(b)). It has been reported that tight junction proteins crosslink to the actin cytoskeleton and form the gut integrity [9]. In addition to alcohol and its metabolites, alcohol-mediated microbial proliferation and LPS have been found to disrupt tight junction proteins and increase intestinal permeability, subsequently intestinal LPS enters blood circulation through broken intestinal barrier [33, 34]. In the present study, we found that high-dose GRg1 effectively reduced the intestinal and circulating levels of LPS and increased the levels of the tight junction proteins in alcohol-treated mice (Figures 7(c), 7(e), and 7(f)). The results indicate that GRg1 pretreatment restores the expression of tight junction proteins and enhances gut barrier by reducing alcohol-induced LPS in the intestine and circulation. IgA restricts the invasion of pathogens and toxins through the mucosa and serves as the major factor of intestinal mucosal defense [35]. We found that GRg1 pretreatment improved the levels of antimicrobial peptides and IgA (Figure 7(d)). Taking together, the findings demonstrate that GRg1 enhances gut barrier function and decreases the gut permeability in alcohol-treated mice.

It is well known that LPS is a microbe-derived bacterial product, which is a crucial mediator of inflammation in ALD. Further studies have reported that alcohol intake increases intestinal permeability, and LPS is transported from intestine to liver through blood circulation, which induces inflammatory response in the liver [36, 37]. The increase of inflammatory cytokines contributes to ALD by regulating the gut-liver axis [9]. LPS-induced TLR4/NF-*κ*B activation builds a link between the gut permeability and liver inflammation, which aggravate liver injury [21, 38]. We found that GRg1 pretreatment reversed the elevated levels of LPS, TLR4, and p-NF-*κ*B in alcohol group (Figure 6 and Table 3). These findings indicate that LPS-mediated TLR4/NF-*κ*B activation is reduced by modulation of gut permeability in GRg1 group. Subsequently, GRg1 pretreatment alleviated the increase of inflammatory factors in the liver treated by alcohol (Table 3). Taking together, the data demonstrated that GRg1 alleviated alcohol-induced inflammatory response through suppression of LPS/TLR4/NF-*κ*B pathway and prevented liver damage, which further proved the target predicted by network pharmacology.

Recently, more evidences have displayed that alcohol results in imbalance of intestinal flora, and the interaction between GM and hepatic damage promotes the development of ALD [39, 40]. In this study, all the groups have unique and shared OTUs (Figure 8(d)). GRg1 pretreatment significantly decreased the values of Chao 1 richness and Shannon diversity in alcohol group, indicating the decline of *α*-diversity (Figures 8(a) and 8(b)). In addition, the GM cluster in high dose-GRg1 group and control group was close, indicating the similarity of *β*-diversity (Figure 8(c)). Our data imply that GRg1 pretreatment can alter GM profile in alcohol-treated mice. Bacteroidetes and Firmicute are the richest phyla, and changes in Firmicutes/Bacteroidetes (F/B) are often associated with health benefits [41, 42]. Our data demonstrated that GRg1 pretreatment enhanced the proportion of Bacteroidetes and Verrucomicrobia and reduced the value of F/B in alcohol group, which were consistent with previous report (Figure 8(e)). *Akkermansia* muciniphila, a gram-negative bacterium located in the mucus layer, maintains the integrity of intestinal barrier [43]. It has been demonstrated that *Bacteroides*, *Lachnospiraceae_NK4A136 _group*, and *Alloprevotella* produce short-chain fatty acids, which protect intestinal barrier function and inhibit inflammatory response [44–46]. Our data showed that *Akkermansia*, *Bacteroides*, *Lachnospiraceae_NK4A136_group*, and *Alloprevotella* were the main microbiota at genus level, and GRg1 pretreatment increased these abundances (Figure 8(f)). In addition, there was specific differences of microbiota between alcohol group and high dose-GRg1 group by LEfSe analysis (Figures 8(g) and 8(h)). Collectively, these findings indicate that GRg1 can regulate GM composition to ameliorate alcoholic liver damage.

It has been demonstrated that excess ethanol intake is correlated with changes in GM composition, intestinal barrier function, and inflammatory response [47]. Some gut bacteria products such as LPS reach the liver through portal vein, which activate TLR4-mediated NF-*κ*B pathway and produce proinflammatory cytokines in the liver [25]. Moreover, alcohol enhances gut leakiness by inhibiting tight junction protein expression and increases LPS load and liver pathology [48]. The interaction between GM alteration and gut-derived inflammation promotes a crucial effect in progression of alcoholic liver injury [9]. Our results demonstrate that inflammatory biomarkers (LPS, TLR4, NF-*κ*B, and cytokines) in the liver showed a negative relation with some microbiota such as Bacteroidetes, Verrucomicrobia, *Akkermansia*, and *Bacteroides* and positive association with Firmicutes after GRg1 pretreatment (Figure 9). The findings demonstrate that LPS-mediated TLR4/NF-*κ*B activation is decreased by alteration of GM in GRg1-treated mice. In contrast, gut barrier defense (tight junction protein expression and sIgA level) exhibited the opposite correlation with these GM. Collectively, our data imply that GRg1 alters the GM, which interacted with gut-derived inflammation, and further alleviates alcoholic liver damage.

## 5. Conclusion

In this study, network pharmacological analysis showed that 11 potential targets of GRg1 against ALD were obtained and implicated with TLR/NF-*κ*B signaling pathways. Meanwhile, GRg1 reduced liver pathological damage and the activities of hepatic enzymes in alcohol-treated mice. GRg1 alleviated alcohol-induced oxidative stress by downregulating oxidative levels and upregulating antioxidative levels. GRg1 inhibits LPS/TLR4/NF-*κ*B signaling pathway, which subsequently ameliorates liver inflammation induced by alcohol. Furthermore, GRg1 inhibited intestinal and circulating LPS levels and increased tight junction proteins and IgA levels, which strengthened the intestinal barrier. GRg1 regulated intestinal flora disturbance, Verrucomicrobia, Bacteroidetes, *Akkermansia*, and *Bacteroides* were positively correlated with intestinal barrier indicators and negatively associated with LPS-mediated inflammation after GRg1 treatment. Our findings proved that GRg1 as a natural product can protect against alcohol-induced liver damage *via* regulating gut-liver axis.

## Figures and Tables

**Table 1:** Primer sequences used for RT-qPCR assay.

Genes	Forward (5′-3′)	Reverse (5′-3′)
GAPDH	ATTCAACGGCACAGTCAAGG	GCAGAAGGGGCGGAGATGA
ZO-1	ACTCCCACTTCCCCAAAAAC	CCACAGCTGAAGGACTCACA
Occludin	CTGTCTATGCTCGTCATCG	CATTCCCGATCTAATGACGC
Claudin-1	GTTTGCAGAGACCCCATCAC	AGAAGCCAGGATGAAACCCA

**Table 2:** The potential targets and network degrees.

Target	Description	Degree
IL-6	Interleukin-6	10
TLR4	Toll like receptor 4	10
TNF	Tumor necrosis factor	10
CASP3	Caspase 3	9
IL-1*β*	Interleukin-1 beta	9
TGF-*β*1	Transforming growth factor-beta 1	9
VEGFA	Vascular endothelial growth factor A	9
BDNF	Brain derived neurotrophic factor	7
CDH1	Cadherin 1	7
NF-*к*B1	Nuclear factor-kappa B subunit 1	7
LPL	Lipoprotein lipase	3

**Table 3:** Inflammation parameters in liver of mice.

Group	Control	Alcohol	GRg1 (10 mg per kg b.w.)	GRg1 (40 mg per kg b.w.)
LPS (EU/g prot)	129.74 ± 7.77	203.41 ± 19.61^###^	198.33 ± 25.67	167.42 ± 13.56^∗∗^
TNF-*α* (pg/mg prot)	166.69 ± 4.58	227.81 ± 7.31^###^	186.64 ± 6.93^∗∗^	171.91 ± 2.96^∗∗∗^
IL-1*β* (pg/mg prot)	20.47 ± 1.93	29.12 ± 1.11^##^	26.84 ± 1.91	24.10 ± 0.49^∗∗^
IL-6 (pg/mg prot)	20.02 ± 1.9	33.69 ± 1.71^###^	28.12 ± 1.85^∗^	23.94 ± 1.24^∗∗^
TGF-*β*1(pg/mg prot)	20.24 ± 1.87	33.22 ± 1.86^##^	27.52 ± 2.66^∗^	24.76 ± 1.93^∗∗^

All data are expressed as mean ± S.D. ^##^*P* < 0.01 and ^###^*P* < 0.001 vs. the control group, ^∗^*P* < 0.05, ^∗∗^*P* < 0.01, and ^∗∗∗^*P* < 0.001 vs. the alcohol-treated group.

## Data Availability

All data supporting the conclusions of this manuscript are provided in the table and figures. Please contact the authors for data requests.

## Abbreviations

AKP: Alkaline phosphatase
ALD: Alcoholic liver disease
ALT: Alanine aminotransferase
AST: Aspartate aminotransferase
b.w.: Body weight
CAT: Catalase
F/B: Firmicutes/Bacteroidetes
GM: Gut microbiota
GRg1: Ginsenoside Rg1
GSH: Glutathione
GSH-Px: Glutathione peroxidase
IgA: Immunoglobulin A
IL-1*β*: Interleukin-1 beta
IL-6: Interleukin-6
LDH: Lactate dehydrogenase
LPS: Lipopolysaccharide
MDA: Malondialdehyde
NF-*κ*B: Nuclear factor-kappa B
p-NF-*κ*B p65: Phospho-nuclear factor-kappa B p65
ROS: Reactive oxygen species
SOD: Superoxide dismutase
TGF-*β*1: Transforming growth factor-*β*1
TLR4: Toll-like receptor 4
TNF-*α*: Tumor necrosis factor alpha
ZO-1: Zonula occludens-1
4-HNE: 4-Hydroxynonenal
8-OHdG: 8-Hydroxy-2′-deoxyguanosin.

## Supplementary Materials

Supplementary Materials Figure S1: the schematic diagram of animal experimental protocol.
