# Supplementary figures and images for: Hepatoprotective Mechanism of Ginsenoside Rg1 against Alcoholic Liver Damage Based on Gut Microbiota and Network Pharmacology
Source: Oxid Med Cell Longev. 2022 Aug 23;2022:5025237. doi: 10.1155/2022/5025237 (PMC9427247; doi:10.1155/2022/5025237)

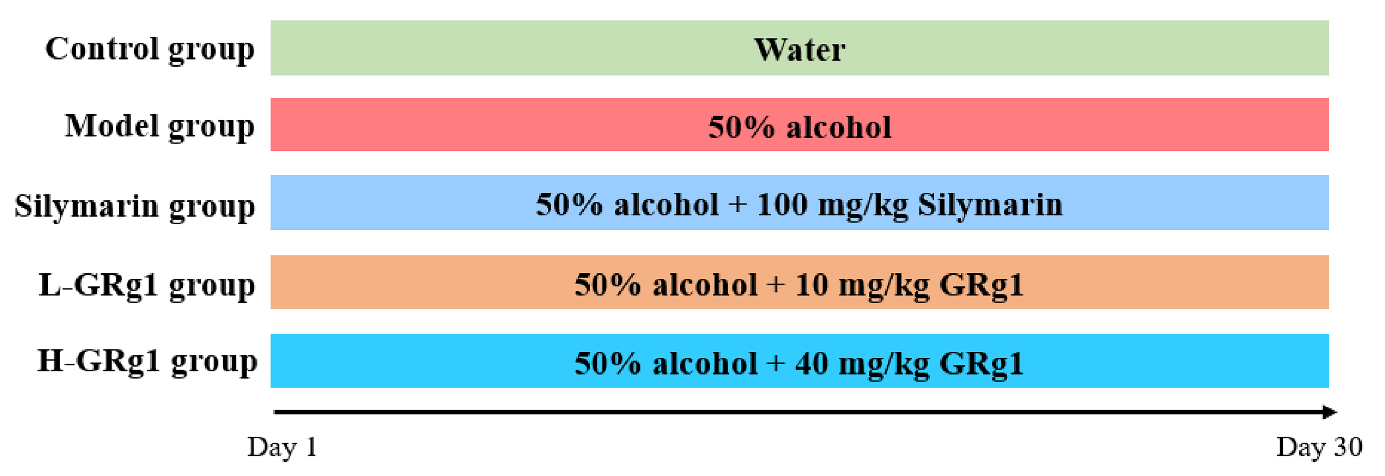


Figure.S1

**Fig. S1** The schematic diagram of animal experimental protocol.

Supplement: Supplementary Materials — Figure S1: the schematic diagram of animal experimental protocol. [file 5025237.f1.docx]
